# A knowledge, attitudes, and practices study on ticks and tick-borne diseases in cattle among farmers in a selected area of eastern Bhutan

**DOI:** 10.1371/journal.pone.0247302

**Published:** 2021-02-22

**Authors:** Jamyang Namgyal, Tenzin Tenzin, Sylvia Checkley, Tim J. Lysyk, Sangay Rinchen, Ratna B. Gurung, Sithar Dorjee, Isabelle Couloigner, Susan C. Cork

**Affiliations:** 1 Department of Livestock, District Veterinary Hospital, Ministry of Agriculture and Forests, Trashigang, Bhutan; 2 Department of Ecosystem and Public Health, Faculty of Veterinary Medicine, University of Calgary, Alberta, Canada; 3 Department of Livestock, National Centre for Animal Health, Ministry of Agriculture and Forests, Thimphu, Bhutan; 4 Khesar Gyalpo University of Medical Sciences of Bhutan, Thimphu, Bhutan; 5 Department of Geography, University of Calgary, Alberta, Canada; University of Lincoln, UNITED KINGDOM

## Abstract

Livestock farming plays an important role in supporting the livelihood of resource-poor subsistence farmers in Bhutan. However, ticks and tick-borne diseases (TBDs) are one of the major constraints to livestock farming due to their negative effect on health and production. To date, no study has been conducted in Bhutan to assess farmers’ knowledge, attitude, and practices (KAP) about ticks and TBDs in cattle, although such information is essential in ensuring the development and adoption of effective prevention and control measures. Therefore, a KAP survey was conducted among 246 cattle owners in the Samkhar sub-district of eastern Bhutan in June 2019, using a structured questionnaire. Based on our scoring criteria, 52% [95%CI: 45.5–58.4] had adequate knowledge about ticks as potential vectors of diseases. Logistic regression analysis showed that the individuals who practiced a stall-feeding system of cattle rearing were 2.8 times [OR = 2.8 (95%CI: 1.66–4.78)] more likely to have adequate knowledge than others. Sixty-eight percent [95%CI: 62.5–74.4] had a favorable attitude toward tick prevention and control programs. Men were 1.95 times [OR = 1.95 (95%CI: 1.09–3.55)] more likely to have a favorable attitude than women, and the individuals who practiced a stall-feeding system were 2.59 times [OR = 2.59 95%CI: 1.45–4.78)] more likely to have a favorable attitude than others, after adjusting for the effect of other variables in the model. Overall, only 38% [95%CI 32.5–45] of the respondents reported tick infestation as one of the most important animal health problems, but 100% reported using acaricides to control ticks in cattle. Despite a high level of acaricide usage, the level of knowledge was low among the farmers interviewed. Findings from this study underline the importance of considering identified knowledge gaps and initiating education efforts to improve the adoption of effective tick prevention and control measures among farmers.

## Introduction

Bhutan is a small Himalayan Kingdom in South Asia located between China to the north, and the Indian states of Assam and West Bengal to the south, Arunachal Pradesh to the east, and Sikkim to the west. It is primarily an agrarian country with 62.2% of the population depending on agriculture and livestock farming for their livelihood [1]. Among all the livestock species reared in the country, cattle (*Bos taurus taurus* L. and *Bos taurus indicus* L.) make the biggest contribution to income and food security in rural communities [2]. Traditionally, the cattle rearing system in Bhutan was categorised into: a transhumant system in the high-altitude areas dominated by cattle migration; and a sedentary system in other areas characterized by crop-cattle integration [3]. Currently, the predominant cattle rearing system is gradually shifting from the traditional free-range systems of the past to a modern stall-feeding system.

Livestock farming, particularly cattle rearing, in Bhutan is constrained by a high burden of infectious diseases such as foot and mouth disease, hemorrhagic septicemia, black quarter, anthrax, rabies, brucellosis, and parasitic diseases [4]. Besides, tick infestation and its associated impact are considered to be one of the major production limiting parasitological problems faced by cattle farming communities [3]. Ticks are not only capable of transmitting infectious agents to livestock but can also directly affect the host due to skin irritation from attachment, blood loss, bite wounds, and sometimes leading to self-trauma and secondary bacterial infections [5]. Heavy infestations can result in anemia and significant weight loss [6]. Some ticks can produce toxins leading to toxicosis and subsequent tick paralysis [7]. In domestic animals, ticks transmit a wide range of diseases, the most important of which are anaplasmosis, babesiosis, cowdriosis, and theileriosis [5, 6]. In Bhutan, three tick-borne diseases (TBDs), anaplasmosis, babesiosis, and theileriosis, are present in cattle, especially in the southern subtropical areas [3] but it is difficult to estimate the actual number of cases due to the limited use of confirmatory diagnostic tests, poor surveillance, and discrepancies in recorded data.

In the public health setting, there is a high incidence of acute undifferentiated febrile illnesses (believed to be caused by rickettsial organisms) reported in Bhutan [8]. The first serological study for rickettsial organisms conducted in 864 persons in Bhutan has found the seroprevalence as follows: scrub typhus group (22.6%); spotted fever group (15.7%); Q fever (6.9%); and typhus group (3.5%) [9]. Scrub typhus in Bhutan is thought to be transmitted by the “chiggers” mite that is endemic in the Himalayan region [8, 10]. Currently, there is no information on the role of ticks in the transmission of rickettsial diseases in Bhutan. However, considering that many rickettsiae are maintained and transmitted by ticks [11], they could be potential vectors for rickettsial organisms in Bhutan [9], and therefore further work is required to understand the burden of TBDs in cattle and humans.

Veterinary services and therapeutics, such as drugs and vaccines, are provided free of charge in Bhutan by the government. Tick control is also a government-supported program, and it is implemented through the Department of Livestock (DoL). The National Centre for Animal Health (NCAH) under DoL is responsible for the selection, procurement, and supply of acaricides in the country. The liquid formulation of pyrethroid compounds (*i*.*e*., cypermethrin, deltamethrin, and flumethrin) and amidines (*i*.*e*., amitraz) imported from India are supplied to farmers for direct topical application to host animals [12]. Livestock officials advise farmers to follow manufacturers’ instructions during on-farm dilution. These chemicals are preferred because of their broad spectrum of activity against ectoparasites and their mode of action (*i*.*e*., they act by contact), which makes their usage easy and convenient [12]. Data from NCAH showed that in 2019 alone, 42% of the cattle population in Bhutan were reportedly treated for tick infestation, and this cost the government approximately 3.18 million Bhutanese Ngultrum (1USD = Nu.70) for purchasing acaricides. The widespread and indiscriminate use of acaricides has the potential to result in acaricide resistance especially in one-host ticks like *R*. *microplus* (Canestrini) and to cause environmental pollution such as contamination of ground water [13]. However, in Bhutan, no studies have been conducted to evaluate acaricide resistance and environmental impacts.

Besides this conventional method of tick control using acaricides, there has neither been any concerted effort to develop a more effective, sustainable and integrated control strategy, nor an evaluation of the effectiveness of the current tick control methods. The success of tick control programs largely relies on developing a good understanding of farmers’ knowledge about ticks and TBDs, their perceptions of the effectiveness of the proposed control methods, and the socio-cultural context in which such programs are to be implemented [14, 15]. Such information is typically gathered using the most popular and widely used knowledge, attitude, practices (KAP) survey [16]. Although KAP surveys are criticized for extrapolating their data to a wider population for planning purposes [16, 17] yet in the field of ticks and TBDs, KAP studies [14, 15, 18–21] have contributed in the development of effective intervention strategies.

However, in Bhutan, there have not been any KAP studies conducted about ticks and TBDs. Therefore, this KAP study was conducted in the Samkhar sub-district in eastern Bhutan with the primary objective to generate baseline data about knowledge, attitude and practices of farmers regarding ticks and TBDs in cattle, and subsequently develop evidence-based tick prevention and control strategies. Findings from this study are also expected to guide community-based awareness programs about ticks and TBDs in the study area to improve the adoption of effective tick prevention and control measures in cattle.

## Materials and methods

### Study area

**Fig 1:**
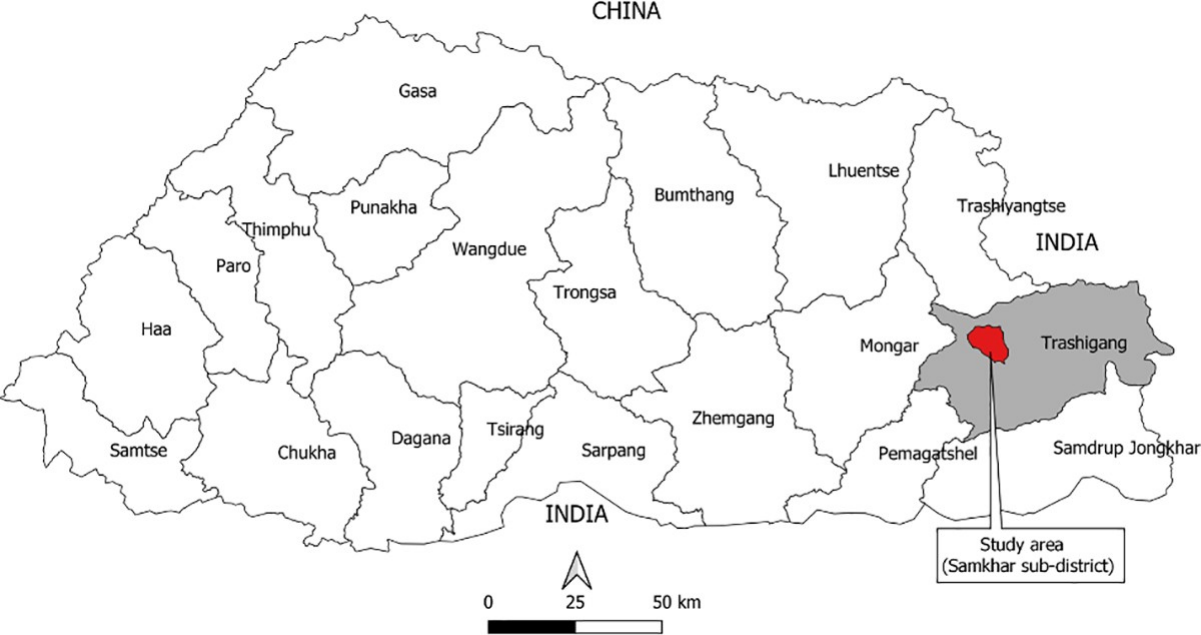
Map of Bhutan showing the study area (Samkhar Gewog) in Trashigang. The names and district boundaries of Bhutan are indicated on the map. The map was prepared using Quantum GIS, QGIS Development Team (2019), QGIS Geographic Information System, Open-Source Geospatial Foundation Project (http://qgis.osgeo.org) and was not taken from another source.

Bhutan is divided administratively into 20 districts (Dzongkhags) and 205 sub-districts (Gewogs). Bhutan’s 20 districts are broadly grouped into the four developmental regions; eastern region, east central region, western region, and west central region. The KAP survey was conducted in the Samkhar Gewog in Trashigang district, eastern Bhutan (Fig 1). The study area was selected based on convenience and purpose [22]. The convenience was that the Regional Livestock Development Centre of the eastern region is in Trashigang from where it was logistically convenient to solicit support during the fieldwork. The purpose was to conduct the study in the most progressive dairy farming areas in the eastern region (*i*.*e*., Samkhar Gewog). The Gewog has a population of 2109 persons living across 62 villages [23], and the cattle population of 2022 in 632 households [24]. There is one livestock extension center managed by a para-veterinarian who provides basic veterinary services.

### Sample size

The sample size of 246 households was calculated using the formula $n=\frac{Z^{2}p(1-p)}{e^{2}}$, where Z(95% level of confidence) = 1.96, p = estimated baseline proportion of cattle owners who were presumed to have adequate knowledge about ticks and TBDs in cattle = 0.20, and e = margin of error = 0.05. Since no previous study was conducted in this selected area, we assumed 20% of the cattle owners would have adequate knowledge of ticks and TBDs in cattle based on the expert opinion of the livestock officials in the Samkhar Gewog. The official list of the households owning cattle in Samkhar Gewog maintained by the District Veterinary Hospital, Trashigang, was used as the sampling frame. Then the simple random sampling in MS Excel 2016 (Microsoft Excel 2016, Redmond, USA) was used to select 246 households required for the study. However, during the questionnaire survey, some selected households could not participate due to reasons such as not owning cattle any more or sociocultural incidents like the death of a family member in the household. In such cases, the nearest household fulfilling the study criteria was selected. Twelve households were replaced.

### Questionnaire survey

A 44-item structured questionnaire consisting of four different sections was prepared in English and used for the collection of data (S1 Questionnaire). Section one consisted of questions on sociodemographic and farming characteristics. Sections two, three, and four consisted of questions on knowledge, attitude, and practices regarding ticks and TBDs in cattle, respectively. Six livestock personnel working in Trashigang district were selected and trained as survey enumerators. Four local government officials assisted the enumerators in identifying the households during the survey. The questionnaire was pre-tested in 20 households of Rangshikhar village through mock interviews that were part of the survey enumerators’ training. Based on the pre-test, modifications were made to the questionnaire to suit the local context.

The two inclusion criteria were households owning cattle and respondents aged not less than 18 years. This study targeted household heads or any senior member of the family, who usually reside in a household throughout the year, to be the main respondents as they were usually directly responsible for management of cattle. Before starting the questionnaire survey, the enumerators explained the objective of the study to the selected respondents, and verbal consent was sought for the interview. Respondents were informed that participation was voluntary and that they could withdraw anytime during the interview. All the selected respondents agreed to participate in the interview. The face-to-face interviews were conducted in June 2019 in local dialects but recorded in English. Data collection was conducted using a web-based mobile phone application, EpiCollect5 (https://five.epicollect.net/).

### Ethics statement

The study protocol was approved by both the Conjoint Faculties Research Ethics Board (CFREB), University of Calgary, Canada (REB19-0035), and the Research Ethics Board of Health (REBH), Ministry of Health, Royal Government of Bhutan (ref no. REBH/PO/2019/029).

### Statistical analyses

The data collected through EpiCollect5 were checked for completeness using data filtering options and imported to R computing software (R Core Team 2018, Vienna, Austria) for analyses. The method used by Tack *et al*. [25] was adapted to categorize the respondents as either having “adequate knowledge” or “inadequate knowledge”. A score was assigned to two questions, and the knowledge was considered adequate when the respondents answered both the questions correctly (S1 Table). Based on the score, the knowledge was converted into a binary outcome variable (*i*.*e*., 1 for respondents who had “adequate knowledge” and 0 for respondents who had “inadequate knowledge”). The assumption was that the respondents with adequate knowledge would be aware that the ticks could transmit diseases to humans and cattle.

Similarly, the attitude was described using the methods of Dhimal *et al*. [26] and Rinchen *et al*. [27] to categorize respondents as either having a “favorable attitude” or “unfavorable attitude”. Three questions were scored to evaluate respondents’ attitudes to tick prevention and control programs (S2 Table). The respondents could choose an answer on a Likert scale of 5 (1: strongly disagree, 2: disagree, 3: no opinion, 4: agree, and 5: strongly agree). The attitude was considered favorable when the responses to all three questions were “agree” and “strongly agree”. Based on the score, the attitude was converted into a binary outcome variable (*i*.*e*., 1 for respondents who had a “favorable attitude”, and 0 for respondents who had an “unfavorable attitude”).

The sociodemographic variables such as age, gender, education level, cattle holding per household, and husbandry practice were considered explanatory variables against each of the binary outcomes of knowledge and attitude variables. For analysis, the variable age was categorized based on the quartile distribution as 18–35, 36–45, and >45 years, and the variable cattle holding per household as above 4 or below 4 based on the mean number of cattle owned by the households sampled. The variable education was categorized as “literate” or “illiterate”, and the variable husbandry practice as “stall feeding” or “mixed practices”. A descriptive analysis was carried out for the entire dataset to calculate frequencies and proportions.

Logistic regression analyses were conducted using the sociodemographic variables as explanatory variables against each of the binary outcome variables of knowledge and attitude (S3 and S4 Tables). The explanatory variables with P-value ≤ 0.25 in univariable analyses were selected for multiple logistic regression analyses [27]. The final multiple logistic regression models were manually built using a forward stepwise selection approach. For each model, first, a variable with the smallest P-value in the univariable analyses was entered into the model. Subsequently, each of the remaining variables was individually added to the model (one at a time) to determine whether its addition improved the fit of the model significantly at P-value ≤ 0.05. A likelihood ratio test was used to select significant variable that had the greatest improvement in the likelihood ratio statistic. Variables no longer associated with the outcome were removed, and only the variables with P-value (P≤0.05) were retained in the final model. Confounding was assessed by adding the variables that were removed from the final model [28]. A variable was to be considered a confounder if it changed the coefficient of the significant variables by more than 25%. Multicollinearity of the predictors in the models was also assessed using the variance inflation factor (VIF) at the cut-off of 2.5 [29]. Interactions were assessed by adding a cross-product term (*i*.*e*., cattle holding*husbandry practice). The odds ratio (OR) and its 95% confidence interval (CI) of the variables associated with the outcome variables were calculated from the final multiple logistic regression models.

## Results

### Sociodemographic characteristics

Two hundred and forty-six respondents were interviewed, and the response rate was 100 percent. The mean age of the respondents was 46 years. The detail of the sociodemographic and farm characteristics is presented in Table 1.

**Table 1:** Sociodemographic characteristics of the respondents.

Variables	Categories	Total (n = 246)	Percentage
**Gender**	Male	100	40.5
	Female	146	59.1
**Age (years)**	18–35	62	25.1
	36–45	61	24.8
	>45	123	50
**Education level**^§^	Not attended any school	158	63.9
	Attended/Attending NFE^£^	54	21.9
	Primary level	15	6.1
	Secondary level	13	5.3
	Buddhist studies^€^	6	2.4
**Cattle holding per household**	Above or equal to 4	165	66.8
	Below 4	81	32.8
**Husbandry practice**^¥^	Mix of stall feeding & tethered grazing	110	44.5
	Mix of stall feeding & free grazing	30	12.1
	Stall feeding	102	41.3
	All-time free grazing	4	1.6

^§^ The participants under “Not attended any school” were considered “Illiterate”, while the rest were considered “Literate” in the analyses.

^£^ NFE is a non-formal education program in Bhutan targeted toward building literacy in rural communities.

^€^ Buddhist studies refer to either formal or non-formal education imparted by Buddhist monasteries.

^¥^ The participants under “Stall feeding” were considered “Stall feeding”, while the rest were considered “Mixed practices”.

### Knowledge about ticks and TBDs in cattle

All 246 respondents had seen ticks on the cattle: 106 (43.1%) had also seen them on vegetation in the forests; 11 (4.5%) had seen them on cattle and in pasturelands; and 8 (3.3%) on cattle and in agricultural fields too. The majority, 131 (53.3%) of the respondents, were not aware of how cattle became infested, while 103 (41.9%) identified the forest as the source, and 12 (4.8%) identified grazing land, fodder grasses, and bedding materials as the other sources of ticks. One hundred and sixty-eight respondents (68.3%) reported that the ticks were commonly seen in summer (June, July, and August), while 24 (9.8%) reported having seen them in winter (December, January, and February). However, 54 (21.9%) reported that ticks were seen throughout the year. One hundred and twenty-two respondents (49.6%) reported that ticks were commonly found in warm places, while 29 (11.8%) reported finding them in cold places. However, 95 (38.6%) reported having found ticks in both warm and cold places. In Bhutan, warm places are areas characterized by subtropical climate with temperature ranging from 17.2 to 23.6°C; while cold places are those characterized by temperate and alpine climate with temperature ranging from 5.5 to 12.5°C [30].

The majority of the respondents (134, 54.5%) believed that the European breeds of cattle were more susceptible to tick infestation, 80 (32.5%) thought the indigenous breeds were more susceptible, and 32 (13%) responded “Don’t know”. Most of the respondents (152, 61.8%) thought old cattle to be the most affected by tick infestation, 35 (14.2%) thought young cattle, 31(12.6%) thought adult cattle, 13 (5.3%) thought heifers, and 16 (6.5%) responded “Don’t know”. With regard to predilection sites of ticks on the body of animals, neck and groin regions were considered to be the most common sites by 233 (94.7%) and 190 (77.2%) of the respondents, respectively. One hundred and sixteen (47.2%) thought that ticks stay on the body of the animals unless removed, 111 (45.1%) reported that the ticks would drop off from the body of animals after the blood meal, and 19 (7.7%) responded “Don’t know”.

When asked about the general health and production impacts of tick infestation in cattle; weight loss was mentioned by 239 (97.2%) of the respondents, blood-sucking by 191 (77.6%), production loss by 155 (63%), bite wound by 144 (58.5%), anorexia by 32 (13%), hide damage by 7 (2.8%), red/brown color urine by four (1.6%), and fever by just one (0.4%). Most respondents (156, 63.4%) considered ticks to be potential vectors of TBDs in cattle; however, 242 (98.4%) indicated that they had never heard of any particular names of TBDs in cattle—indicating that many of them just know in general about the health and production impacts of tick infestation in cattle. One hundred and forty-eight respondents (60.2%) experienced tick bites at one point in their lives. However, 90 (36.5%) of the respondents incorrectly believed that the tick bites would not transmit diseases to humans. Pain and irritation as symptoms of tick bites were reported by 147 (99.3%) respondents out of 148 who experienced tick bites themselves, rash and swelling around the bite site by 111 (75%), fever and headache by 15 (10.1%), and “no symptom” by one respondent.

**Fig 2:**
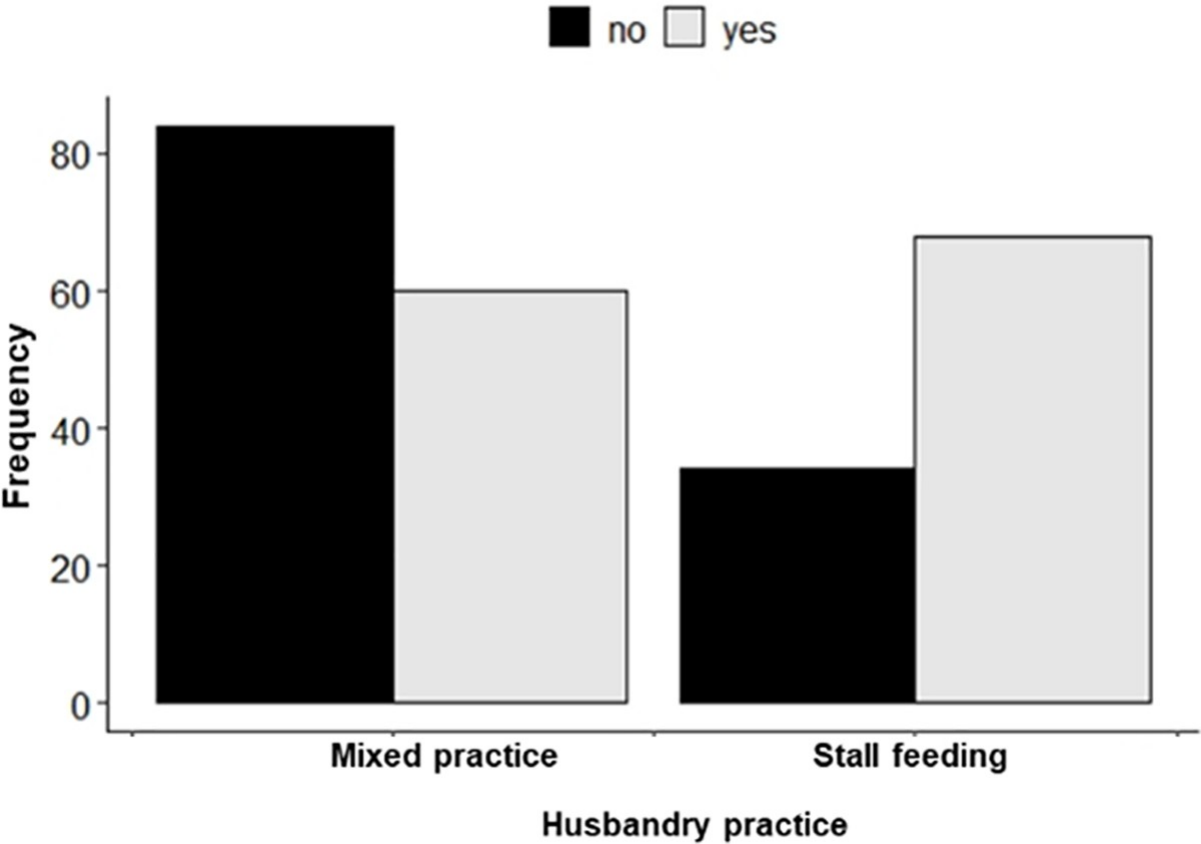
Respondents who had “adequate vs. inadequate knowledge about ticks as potential vectors of diseases” categorized by husbandry practice (n = 246).

The analysis of the knowledge score showed that out of 246 respondents, 128 (52%) [95%CI: 45.5–58.4] had adequate knowledge about ticks as potential vectors of diseases in humans and animals (Fig 2). The multiple logistic regression analysis showed that husbandry practice was the only variable significant in the final model. Individuals who practiced the stall-feeding system of cattle rearing were 2.8 times [OR = 2.8 (95%CI: 1.66–4.78)] more likely to have adequate knowledge about ticks as potential vectors of diseases than that of others who had a mixed practice of cattle rearing (Table 2). No confounding variable, outliers, and influential observations were found in this analysis. The interaction term (*i*.*e*., cattle holding*husbandry practice) was not significant in this analysis.

**Table 2:** Results of multiple logistic regression analyses to determine the association between the explanatory variables and each of the binary outcome variables of knowledge (having adequate knowledge about ticks as potential vectors of diseases or not) and attitude (having a favourable attitude toward tick control programs or not).

Variables	Categories			Total	Estimate ± SE	Adjusted OR (95%CI)	Overall P-value
		**Adequate knowledge**					
		**Yes**	**No**				
**Husbandry practice**	Mixed practice	84	34	118	-0.336 ± 0.17	reference	<0.001
	Stall-feeding	60	68	128	1.0296 ± 0.27	2.8 (1.66–4.78)	
		**Favorable attitude**					
		**Yes**	**No**				
**Intercept**					0.177 ± 0.209		
**Husbandry Practice**	Mixed practice	88	56	144		reference	0.002
	Stall-feeding	81	21	102	0.954 ± 0.303	2.59 (1.45–4.78)	
**Gender**	Female	93	53	146		reference	
	Male	76	24	100	0.670 ± 0.298	1.95 (1.09–3.55)	

Parameter significant at P < 0.05.

### Attitude toward tick prevention and control programs in cattle

**Fig 3:**
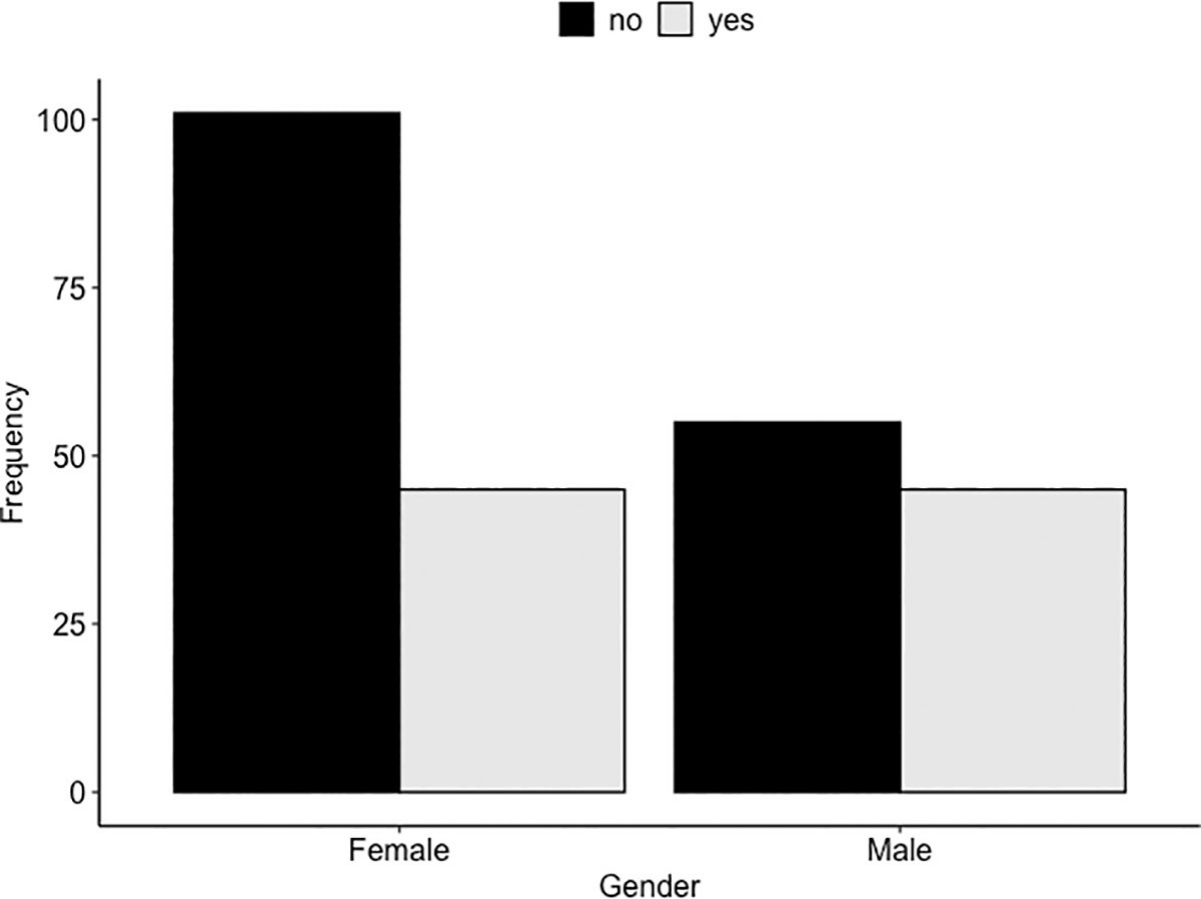
Respondents who had a “favorable vs. unfavorable attitude towards tick control programs” categorized by gender (n = 246).

**Fig 4:**
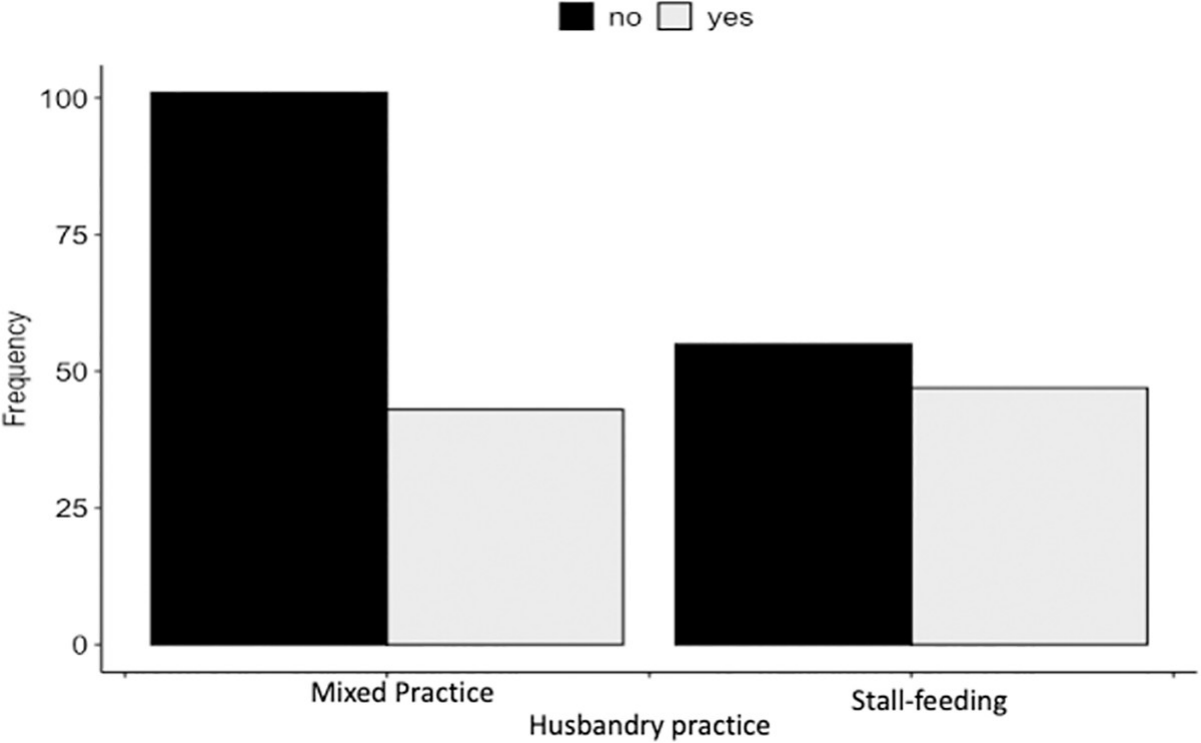
Respondents who had a “favorable vs. unfavorable attitude towards tick control programs” categorized by husbandry practice (n = 246).

Based on our scoring criteria, 169 (68.7%) [95%CI: 62.5–74.4] of the respondents had a favorable attitude toward tick prevention and control programs (Figs 3 and 4). The multiple logistic regression analysis showed that gender and husbandry practice were the significant variables in the final model. Men were 1.95 times [OR = 1.96 (95%CI: 1.09–3.55)] more likely to have a favorable attitude toward tick prevention and control programs than women, when the other variable in the model is held constant (Table 2). The individuals who practiced stall-feeding were 2.59 times [OR = 2.59 (95%CI: 1.45–4.78)] more likely to have a favorable attitude than that of others who followed mixed practices of cattle rearing, when the other variable in the model is held constant (Table 2). No confounding variable, outliers, and influential observations were found in this analysis. The interaction term (*i*.*e*., cattle holding*husbandry practice) was not significant in this analysis.

### Self-reported farm practices

Of the 246 respondents: 147 (59.5%) reared cattle for generating income through the sale of milk and dairy products; 77 (31.2%) for family consumption of dairy products; 20 (8.1%) for manure; and 2 (0.8%) for draft purpose. One hundred and ninety-seven respondents (79.8%) had a cattle shed with corrugated galvanized iron (CGI) sheet roofing and concrete flooring, while 40 (16.2%) had a conventional type of shed built out of locally available materials. Only nine (3.6%) practiced open-air tethering. Of the 197 (79.8%) respondents who had improved cattle sheds with CGI sheet roofing and concrete flooring, 189 (96.9%) reported washing floors on a daily basis. Overall, 95 (38.5%) reported using bedding materials in their cattle shed, out of which, 69 (72.6%) reported using leaf litter, 12 (12.6%) reported using bracken fern, 12 (12.6%) reported using corn straw, and 2 (2.1%) reported using paddy straw. Out of 95 respondents who reported using bedding materials, 70 (73.7%) used it in winter, 20 (21.1%) throughout the year, and 5 (5.3%) in summer.

The three most important animal health problems reported in this study were: milk fever that was reported by 159 (64.6%) of the respondents, mastitis by 157 (63.8%), and foot and mouth disease by 109 (44.3%). Only 95 (38.6%) of the respondents reported tick infestation as the most important animal health problem. The three main purposes of visiting livestock centers were: “to receive acaricide” that was reported by 244 (99.2%); “to receive medicine” by 239 (97.2%); and “to receive deworming drugs” by 175 (71.1%). All 246 respondents (100%) reported using acaricides for controlling ticks in cattle. During the peak infestation season, 147 (59.8%) of the respondents reported having used acaricides occasionally; 32 (13.8%) on a monthly basis; 43 (17.5%) on a fortnightly basis; and 24 (9%) on a weekly basis. While applying acaricides to host animals, 241 (98%) of the respondents reported having followed hand dressing method while 5 (2%) followed hand spraying. To obtain basic information on the efficiency of the acaricides used, the respondents were asked about how long it took for the acaricides to cause ticks to drop off from the body of host animals. One hundred and five respondents (42.7%) reported that ticks dropped off within a day, 81 (32.9%) within a few hours, 58 (23.6%) within a few days, and 2 (0.8%) within a week. Regarding the management of dropped off ticks: 133 (54.1%) of the respondents reported to have done nothing, 99 (40.2%) reported to have flushed them away with water, 11 (4.5%) reported to have collected and thrown them into the field, and 3 (1.2%) reported to have collected and burned.

Ninety (36.6%) of the respondents reported to have “always” checked their body for ticks after handling tick-infested cattle, 91(36%) reported to have checked “sometimes”, and 65 (26.4%) never checked. Similarly, after visiting the forests, 85 (34.6%) reported to have “always” checked their body, 101(41.1%) reported to have checked “sometimes”, and 60 (24.4%) never checked. When the veterinary centers had no acaricides, the respondents reported having followed the mixed practices of manual removal and indigenous medicine to control ticks on cattle. Of these methods: 136 (55.3%) followed manual removal, 66 (26.8%) applied *Zanthoxylum* solution, 55 (22.4%) brushed animals, 38 (15.4%) applied salt solution, and 61 (24.8) reported to have done nothing. To determine the farmers’ awareness of acaricides’ properties, a question was asked if acaricides can be used for purposes other than treating tick infestation. One-hundred twenty-four respondents (50.4%) reported that they did not know about any other use; however, 122 (49.6%) reported that it could be used as either a pesticide (for crops) or an insecticide (at homes).

## Discussion

To our knowledge, this is the first KAP study conducted to determine livestock farmers’ knowledge, attitudes, and practices about ticks and TBDs in cattle in Bhutan. In this study, all respondents reported having seen ticks. This is not surprising given that the respondents were farmers whose lives are intimately linked with animals, pastures, and forests, where ticks are commonly found. Despite all respondents having seen ticks, more than half of the respondents did not know how cattle become infested and where ticks are commonly found in the environment. Some respondents thought that ticks are more common in winter. Normally, low temperatures in winter are likely to slow down the developmental processes of ticks as the processes such as molting, oviposition, and questing are dependent on temperature [31]. Ticks are known to quest for hosts only at temperatures greater than 7°C [32]. In the neighboring Indian state of West Bengal, ticks are more common in monsoon season and summer than in winter [33]. However, winter in Bhutan is normally dry and cold with the mean temperature of 15°C, and the fodder resources also become very scarce. Most cattle lose their body condition and immune capacity to build resistance to ticks, predisposing many to tick infestation. This could explain why some respondents thought ticks were more common in winter.

Generally, indigenous breeds of cattle are considered to be highly resistant to ticks, and they are known to be reared with minimum tick control by exploiting their innate immunity [3, 5, 34]. While more than half of the respondents recognized indigenous breeds of cattle as generally resistant to ticks, there were some respondents who reported a belief that indigenous breeds of cattle are the most affected. This inconsistency in the beliefs could be due to existing differences in the systems of rearing cattle between the European and indigenous breeds. The latter are mostly reared in a free grazing system, where the animals spend most of their time in the forests and pasturelands [35], getting more frequently exposed to ticks. Unlike stall-fed European cattle, management practices like grooming and brushing are rarely practiced in the indigenous breeds resulting in heavy tick infestation. Therefore, their physical appearance (with a lot of ticks on their body) gives an impression that they are more susceptible. Among the age groups, old cattle and young calves were reported to be the most affected, which is in agreement with the findings from studies conducted in the Indian state of West Bengal [33], Bangladesh [36], Ethiopia [37], and Nigeria [38]. The reason for higher tick infestation in these two age groups is attributed to underdeveloped immunity in young and weak immunity in old cattle. Moreover, unlike the productive adult cattle that are given the utmost managemental care, the young calves and old cattle are the least attended. They can, therefore, act as a reservoir for ongoing environmental tick contamination.

Most of the respondents provided accurate and detailed clinical descriptions of tick infestation in cattle, but there were only four respondents who reported brown-colored urine (hemoglobinuria), and one respondent who reported observing fever. Generally, these two signs are typical for babesiosis in cattle [39]. The fact that only four and one respondents reported hemoglobinuria and fever, respectively, is an indication that the majority of the farmers could not relate clinical signs such as hemoglobinuria and fever to ticks and TBDs. Furthermore, hemoglobinuria is often confused with hematuria, which is commonly associated with bladder tumors linked to chronic bracken fern poisoning in Bhutan [40].

Despite the presence of TBDs such as babesiosis and theileriosis in the study area, there has been no recent effort made from veterinary laboratories to diagnose and record cases in a systematic manner due to a shortage of manpower and resources. The prevailing practice followed with regard to such TBDs is treating the animals based on clinical signs. Moreover, since there was no major outbreak resulting in mortality of animals due to such TBDs, the farmers, as well as the veterinary officials, had no reason to be concerned. Further, the immunity acquired through previous exposure(s) to ticks makes the cattle “endemically stable” [5]. It was observed that a large proportion of the respondents had never heard of any particular names of TBDs in cattle. This could have been due to the lack of awareness programs on ticks and TBDs in recent years by livestock officials which is likely due to limited resources in the absence of any major outbreak.

According to our criteria, 52% of the respondents had adequate knowledge of ticks as potential vectors of diseases. The multiple logistic regression analysis showed that the farmers practicing the stall-feeding system of cattle rearing were more likely to have adequate knowledge than that of others following mixed practices of cattle rearing. This suggests the positive impact of the Royal Government of Bhutan’s livestock intensification program that promotes the stall-feeding system [35]. In this system, the primary focus is to enhance the health and productivity of cattle and subsequently improve rural livelihood through cash income generated from the sale of milk and dairy products. The government, through the provision of subsidized livestock inputs such as the purchase of high-yielding cows, shed construction materials, feed and fodder, and farm and marketing equipment, encourages as many farmers as possible to take up the modern market-based farming. Training and awareness programs on clean milk production, livestock health management, crossbreeding, fodder conservation, and so on are also provided regularly. As a result, farmers interact more with livestock officials and avail their technical support services. These interactions would have contributed to equipping farmers with some degree of knowledge about the potential role of ticks as vectors of diseases.

Overall, 68% of the respondents had a favorable attitude toward tick prevention and control programs. The observation that men had a more favorable attitude than women could not be strongly associated with any social factor. However, a person’s knowledge, beliefs, emotions, and values, are closely interlinked with attitudes, which can either be positive or negative [16]. Therefore, a key factor could be that the men get more opportunities to attend government-initiated meetings and training programs leading to men having better knowledge about ticks and TBDs than women. However, this trend has been gradually changing, and now women also attend such programs. The positive association between the farmers who were practicing the stall-fed system of cattle rearing and having a favorable attitude can likely be attributed to the adoption of the government’s livestock intensification and commercialization programs that promote the stall-feeding system, which most of the farmers in the study area practice. In this system, animals are normally kept inside the sheds and are rarely let out for grazing. This not only protects the animals from accidental falls and fights with other animals but also reduces their exposure to ticks. In a study in Pakistan [41], tick prevalence has been found to be lower in the farms where the stall-feeding system is practiced. Consequently, such positive outcomes largely influence the farmers’ attitude. Tick infestation is also known to be determined by the types of housing. The odds of acquiring tick infestation are higher in animals housed in poorly constructed sheds lacking proper ventilation [41]. Plastering of floor surfaces and walls with smooth cement also helps avoid shed infestation by removing the potential hiding places of some tick species (such as *Hyalomma*) that hide in cracks and crevices [5]. Most of the cattle sheds in the study area have concrete flooring and CGI sheet roofing, and the floors are washed on a daily basis. This practice could be mitigating the infestation of sheds, but we need to undertake additional studies to ascertain this.

Traditionally, farmers in Bhutan use leaf litter as bedding material in their cattle sheds, especially during the cold winter months (December, January, and February). Leaf litter is not only the source of warmth for animals but also an important component of farm manure. Almost every household in the country was given a legal right to a small block of tree grove known as *Sokshing* for the collection of valuable leaf litter [42]. In our study area, some farmers reported using leaf litter as bedding material, especially in winter, but this practice is declining as the crop-cattle integration system of farming is fast fading. Ticks are known to survive well in leaf litter as it provides consistent insulation from cold conditions of winter [43]. The infestation of cattle in winter can also be associated with the use of leaf litter. Some farmers also reported using bracken fern as the bedding material, and many farmers (during informal conversations) in the study area consider it as one of the sources of ticks.

Milk fever, mastitis, and foot and mouth disease were perceived to be the three most important animal health problems. Meanwhile, tick infestation was rated as the sixth out of seven options. However, in the section where respondents were made to state the three main purposes of visiting livestock centers, “to receive acaricides” was the number one purpose. This inconsistency may be due to the free supply of acaricides from any livestock center in the country. In the similar KAP studies conducted in Tanzania [44] and Benin [14], where farmers had to bear the cost of acaricides, tick infestation and TBDs were considered a major problem in livestock rearing. However, in Bhutan, farmers are provided free acaricides as and when required, and the subsequent application to the animals would remove ticks present on cattle. Therefore, it is possible that they might have never perceived tick infestation as an important animal health problem.

Throughout the world, acaricidal treatment is still one of the most widely used methods for controlling ticks in cattle [5]. In our study, too, all the respondents reported using acaricides on cattle as well as consider it to be the primary method of controlling ticks. The majority of the respondents reported using acaricides occasionally (*i*.*e*., using them whenever the animals are noticed with high tick infestations). These findings are indicative of lacking information on the optimal use of acaricides and effective control strategies. Although advice is being given to farmers to strictly adhere to a specific dilution rate, there is no system to monitor the usage in the field. Effective tick control approaches such as seasonal treatments at the peak of tick activity and intensive treatments at the beginning of the tick season are not followed. This is also due to our livestock officials lacking information on local tick species diversity and life cycles.

Buddhist traditions and culture also influence tick control practices in Bhutan. Every year, the Bhutanese observe *Saga dawa—*the auspicious month, according to the Bhutanese lunar calendar [45]. This auspicious month normally falls sometime in the spring, coinciding with the peak tick season in Bhutan. In this month, most of the Bhutanese avoid non-virtuous and harmful activities, and killing ticks is also considered as a non-virtuous act. Consequently, most of the farmers normally refrain from using acaricides during this month. Our survey coincided with the *Saga dawa* (which happened to have fallen in June that year). Therefore, heavy tick infestation was observed in cattle, as most of the farmers thought that they would apply acaricides once the *Saga dawa* month was over. Such cultural practice should also be taken into consideration as we look ahead to improving tick control strategies.

The efficacy of acaricides on the susceptibility of ticks is assessed by conducting *in vitro* tick immersion assays using acaricide solutions prepared based on manufacturers’ instructions and then evaluating its impact on mortality and egg production by female ticks [46]. To date, there has been no such assessment done to evaluate the efficacy of acaricides used in Bhutan. However, one crude way of assessing the efficacy at the farm level could be observing tick drop-off from the body of an animal after applying acaricides. In our study, the majority of the respondents reported tick drop-off occurring within a day, some reported within a few hours, and a few reported within a few days. While this finding does not indicate anything substantial regarding the efficacy of the acaricides, it suggests that some of the farmers could be using an incorrect on-farm dilution of the acaricides.

Since the acaricide supply in Bhutan is regulated by the government through the Department of Livestock (DoL), it is available only in the livestock centers in the country. When there is a shortage or an inconsistent supply, farmers either practice manual removal or use *Zanthoxylum armatum* DC. seeds as traditional indigenous medicine against ticks. *Zanthoxylum armatum*, commonly known as “Thi-ngye” in Bhutan, is an important medicinal plant widely distributed in subtropical and temperate valleys of the Himalayas, including Bhutan, and it has several ethno-pharmacological uses [47]. The use of *Zanthoxylum* spp. as an acaricide is documented in studies in Pakistan [48] and Brazil [49]. The latter determined the acaricidal properties of its essential oil through an adult immersion test using engorged female ticks. The essential oil (5% concentration) of *Zanthoxylum caribaeum* Lam. caused 65% mortality in day one, 85% in day two, and 100% in day five [49]. In Bhutan, when *Zanthoxylum* is used as an acaricide, the seeds are soaked in water overnight, and the solution is applied to the animals. In spite of such available options, 24.8% of the respondents in the study area reported doing nothing when there was no acaricide in their livestock center. This affirms how important it is for DoL to maintain a consistent supply of acaricides to farmers. Other non-chemical tick control methods such as predators (like backyard poultry), environmental clearing, and rotational grazing [6] may be difficult to practice in Bhutan. Backyard poultry destroy family vegetable crops, environmental clearing affects the environment as well as involves a considerable cost [6], and rotational grazing is not feasible as the individual landholdings are very small [50].

As Bhutan was slowly phasing out the supply of pesticides to farmers to make the country’s agriculture 100% organic, the cross-application of acaricides on the crops was a growing concern among livestock officials. To understand the situation at the farmers’ level, a question was asked if they knew any other use of acaricides besides controlling ticks. Half of the respondents reported not being aware of other purposes, while the other half reported that it could be used either as a pesticide (for crops) or as an insecticide (at homes). Some farmers in the study area also admitted to using leftover acaricides in their vegetable fields. Therefore, if left unregulated, there is a possibility that such incidents might increase over the years.

The main limitation of this KAP study is that it was designed for a specific location (*i*.*e*., targeting the most progressive dairy farming area), and the findings cannot be generalized to other areas with different context and farming systems. However, the findings do provide useful information to assist in the development of education and extension activities that can be used even beyond the study area. Although the interview targeted household heads, during the survey, the enumerators interviewed those available at home. This could have led to some response bias in the answers to our questions. Considering the cultural background in Bhutan, household heads (whether male or female), are closely involved in all farming activities, and so they are likely to provide reliable and accurate information. The findings about “farm practices” could be biased as the responses were self-reported, and also the descriptive data about practices fails to explain why certain practices are chosen.

A bias could have occurred due to language and cultural context (*e*.*g*., questionnaire was prepared in English but the interview was conducted in a local dialect); however, we minimized this potential bias as the enumerators were livestock department officials familiar with the language, culture, and practices of the farmers interviewed. The interpretation bias was reduced as the author has previously worked in the study area and is familiar with the cultures and practices of the farmers in this region. Overall, the findings from this KAP study have contributed to the larger understanding of farmers’ perspectives about ticks and TBDs in cattle in Bhutan, and also provide useful baseline data that future researchers can use to develop further studies. The study has also engaged community members directly and in doing so has already raised awareness about ticks and tick-borne diseases in cattle.

## Conclusions

In this study, only 52% the farmers had adequate knowledge about ticks as potential vectors of diseases. Therefore, awareness programs should focus on informing farmers on topics such as the role of ticks as potential vectors for diseases in animals and humans, the life cycle and seasonal pattern of locally present tick species, effective tick control strategies, and appropriate use of acaricides. This study also observed that the farmers in the study area did not perceive ticks and TBDs as significant problems for livestock health. However, in recent years, there has been a discussion at the policy level about supplying acaricides on a cost-sharing basis. Should the government implement this cost-sharing system, farmers might need to design tick control strategies of their own and will likely want to reduce cost implications. The Department of Livestock (DoL) might have to provide technical support to strategize tick control in such a way that it suits a particular farming system. This is where the findings of this and other KAP studies would play an important role in designing and implementing tick control programs. Therefore, we recommend similar KAP studies in other farming communities in Bhutan.

## Supporting information

S1 Table Questions used for assessing participants’ knowledge about ticks as vectors of diseases. (DOCX)

S2 Table Questions used for assessing participants’ attitude toward prevention and control of ticks in cattle. (DOCX)

S3 Table Results of logistic regression analysis to understand the association between the explanatory variables and the binary outcome variable (having adequate knowledge about ticks as potential vectors or not). (DOCX)

S4 Table Results of logistic regression analysis to understand the association between the explanatory variables and the binary outcome variable (having a favourable attitude toward tick control programs or not). (DOCX)

S1 Questionnaire Questionnaire for assessment of knowledge, attitude, and practices (KAP) survey on ticks and tick-borne diseases among cattle owners in Samkhar gewog, Trashigang, Bhutan. (DOCX)

S1 Dataset Data collected during the survey and used for analysis. (XLSX)
