# Supplementary material for: A knowledge, attitudes, and practices study on ticks and tick-borne diseases in cattle among farmers in a selected area of eastern Bhutan
Source: PLoS One. 2021 Feb 22;16(2):e0247302. doi: 10.1371/journal.pone.0247302 (PMC7899374; doi:10.1371/journal.pone.0247302)
Supplement: S1 Table — (DOCX) [file pone.0247302.s001.docx]

**S1 Table. Questions used for assessing participants’ knowledge about ticks as vectors of diseases.**

| **Questions** | **Score** | **Criteria** |
| --- | --- | --- |
| Do you think cattle can get diseases from ticks? * | 1 | A point was awarded if respondents answered “Yes”; otherwise, no point was awarded. |
| Do you think humans can get diseases from tick bites? * | 1 | A point was awarded if respondents answered “Yes”; otherwise, no point was awarded. |

*If respondents answered any of these questions incorrectly, they were categorized as not having adequate knowledge about ticks as vectors of diseases.
