# Supplementary material for: A knowledge, attitudes, and practices study on ticks and tick-borne diseases in cattle among farmers in a selected area of eastern Bhutan
Source: PLoS One. 2021 Feb 22;16(2):e0247302. doi: 10.1371/journal.pone.0247302 (PMC7899374; doi:10.1371/journal.pone.0247302)
Supplement: S2 Table — (DOCX) [file pone.0247302.s002.docx]

**S2 Table. Questions used for assessing participants' attitudes toward prevention and control of ticks in cattle.**

| **Questions** | **Score** | **Criteria** |
| --- | --- | --- |
| **Do you agree?** Proper use of synthetic acaricides can reduce the incidence of tick infestation in cattle. * | 4&5 | A point was awarded if the answer was "agree" and “strongly agree”; otherwise, no point was awarded. |
| **Do you agree?** The risk of the tick infestation can be reduced by keeping cattle always in a shed. * | 4&5 | A point was awarded if the answer was "agree" and “strongly agree”; otherwise, no point was awarded. |
| **Do you agree?** Adopting good farm practices can reduce the risk of tick infestation (*e.g.*, regular washing of floor, regular checking of animals, avoiding the use of bedding materials. * | 4&5 | A point was awarded if the answer was "agree" and “strongly agree”; otherwise, no point was awarded. |

**If respondents answered any of these questions incorrectly, they were categorized as not having a favorable attitude toward prevention and control of ticks in cattle.
