# Supplementary material for: A knowledge, attitudes, and practices study on ticks and tick-borne diseases in cattle among farmers in a selected area of eastern Bhutan
Source: PLoS One. 2021 Feb 22;16(2):e0247302. doi: 10.1371/journal.pone.0247302 (PMC7899374; doi:10.1371/journal.pone.0247302)
Supplement: S3 Table — (DOCX) [file pone.0247302.s003.docx]

S3 Table. Results of logistic regression analysis to understand the association between the explanatory variables and the binary outcome variable (having adequate knowledge about ticks as potential vectors or not).

| **Variable** | **Intercept ± SE** | **Slope ± SE** | **Z** | **χ^2^** | **P(>χ^2^)** | **OR (95% CI)** | **AIC** | **VIF** |
| --- | --- | --- | --- | --- | --- | --- | --- | --- |
| **Univariable logistic regression analysis** | |  |  |  |  |  |  |  |
| Gender (male) | -0.055 ± 0.166 | 0.337 ± 0.261 | 1.289 | 1.67 | 0.197 | 1.4 (0.84-2.34) | 342.95 |  |
| Age (18-35) | 0.111 ± 0.236 | 0.281 ± 0.350 | 0.802 | 2.47 | 0.423 | 1.55 (0.84-2.91) | 344.15 |  |
| Age (36-53) | 0.111 ± 0.236 | -0.218 ± 0.303 | -0.722 |  |  | 1.1 (0.59-2.0) |  |  |
| Education (Literate) | 0.101 ± 0.159 | -0.056 ± 0.266 | -0.21 | -0.21 | 0.834 | 0.94 (0.56-1.59) | 344.58 |  |
| Cattle number (>4) | -0.163 ± 0.278 | 0.051 ± 0.052 | 0.988 | 0.99 | 0.323 | 1.05 (0.95-1.17) | 343.64 |  |
| Husbandry practice | -0.336 ± 0.169 | 1.029 ± 0.269 | 3.819 | 15.16 | 0.00 | 2.8 (1.66-4.78) | 329.46 |  |
| **Multiple logistic regression** | |  |  |  |  |  |  |  |
| **Model 1** |  |  |  |  |  |  |  |  |
| Intercept | -0.522 ± 0.208 |  | -2.503 | 17.63 | 0.01 |  | 328.9 |  |
| Husbandry practice |  | 1.065 ± 0.273 | 3.908 |  | 0.00 | 2.9 (1.71-4.99) |  | 1.012 |
| Gender (male) |  | 0.424 ± 0.271 | 1.562 |  | 0.118 | 1.5 (0.90-2.61) |  | 1.012 |
| **Model 2*** |  |  |  |  |  |  |  |  |
| Intercept | -0.336 ± 0.169 |  |  |  |  |  |  |  |
| Husbandry practice |  | 1.029 ± 0.269 | 3.819 | 15.16 | 0.00 | 2.8 (1.66-4.78) | 329.46 |  |

* the best fitted model
