# Supplementary material for: A knowledge, attitudes, and practices study on ticks and tick-borne diseases in cattle among farmers in a selected area of eastern Bhutan
Source: PLoS One. 2021 Feb 22;16(2):e0247302. doi: 10.1371/journal.pone.0247302 (PMC7899374; doi:10.1371/journal.pone.0247302)
Supplement: S4 Table — (DOCX) [file pone.0247302.s004.docx]

S4 Table. Results of logistic regression analysis to understand the association between the explanatory variables and the binary outcome variable (having favourable attitude toward tick control programs or not).

| **Variable** | **Intercept ± SE** | **Slope ± SE** | **Z** | **χ^2^** | **P(>χ^2^)** | **OR (95%CI)** | **AIC** | **VIF** |
| --- | --- | --- | --- | --- | --- | --- | --- | --- |
| **Univariable logistic regression analysis** | | |  |  |  |  |  |  |
| Gender | 0.562 ± 0.590 | 0.172 ± 0.291 | 2.032 | 4.26 | 0.039 | 1.8 (1.03-3.23) | 305.51 |  |
| Age (18-35) | 1.808 ± 0.208 | -0.696 ± 0.332 | -2.096 | 4.73 | 0.94 | 0.49 (0.26-0.96) | 307.04 |  |
| Age (36-53) | 1.808 ± 0.208 | -0.443 ± 0.340 | -1.300 |  |  | 0.64 (0.33-1.26) |  |  |
| Education (Literate) | 0.890 ± 0.175 | 0.281 ± 0.284 | -0.990 | 0.97 | 0.324 | 0.75 (0.43-1.32) | 308.79 |  |
| Cattle number | 0.357 ± 0.303 | 0.092 ± 0.059 | 1.557 | 2.55 | 0.110 | 1.1 (0.98-1.24) | 307.22 |  |
| Husbandry practice | 0.452 ± 0.171 | 0.898 ± 0.299 | 3.007 | 9.59 | 0.002 | 2.4 (1.38-4.48) | 300.18 |  |
| **Multiple logistic regression analysis** | | |  |  |  |  |  |  |
| **Model 1*** |  |  |  |  |  |  |  |  |
| Intercept | 0.177 ± 0.209 |  | 0.847 | 14.82 | 0.397 |  | 296.94 |  |
| Husbandry practice |  | 0.954 ± 0.303 | 3.148 |  | 0.002 | 2.6 (1.45-4.78) |  | 1.012 |
| Gender (male) |  | 0.670 ± 0.298 | 2.247 |  | 0.025 | 1.95 (1.09-3.55) |  | 1.012 |
| **Model 2** |  |  |  |  |  |  |  |  |
| Intercept | 0.103 ± 0.320 |  | 0.3212 | 9.65 | 0.748 |  | 319.45 |  |
| Husbandry practice |  | 0.864 ± 0.300 | 2.878 |  | 0.004 | 2.37 (1.33-4.34) |  | 1.006 |
| Cattle number |  | 0.078 ± 0.061 | 1.280 |  | 0.201 | 1.08 (0.96-1.22) |  | 1.006 |
| **Model 3** |  |  |  |  |  |  |  |  |
| Intercept | -0.111 ± 0.336 |  | -0.332 | 16.05 | 0.740 |  | 297.72 |  |
| Husbandry practice |  | 0.918 ± 0.305 | 3.011 |  | 0.003 | 2.5 (1.39-4.62) |  | 1.018 |
| Gender (male) |  | 0.642 ± 0.299 | 2.146 |  | 0.032 | 1.9 (1.07-3.46) |  | 1.015 |
| Cattle number |  | 0.067 ± 0.062 | 1.091 |  | 0.275 | 1.07 (0.95-1.21) |  | 1.012 |
| **Model 4** |  |  |  |  |  |  |  |  |
| Intercept | 0.171 ± 0.318 |  | 0.538 | 6.44 | 0.591 |  | 305.33 |  |
| Gender(male) |  | 0.568 ± 0.292 | 1.944 |  | 0.052 | 1.76 (1.00-3.16) |  | 1.002 |
| Cattle number |  | 0.086 ± 0.060 | 1.443 |  | 0.149 | 1.1 (0.97-1.23) |  | 1.002 |

* best fitted model.
