# Supplementary material for: A knowledge, attitudes, and practices study on ticks and tick-borne diseases in cattle among farmers in a selected area of eastern Bhutan
Source: PLoS One. 2021 Feb 22;16(2):e0247302. doi: 10.1371/journal.pone.0247302 (PMC7899374; doi:10.1371/journal.pone.0247302)
Supplement: S1 Questionnaire — (DOCX) [file pone.0247302.s005.docx]

| **S1 Questionnaire**  **Title: A knowledge, attitudes, and practice (KAP) survey on ticks and tick-borne diseases among cattle owners in Samkhar gewog, Trashigang, Bhutan.** |
| --- |

| Survey No.  (3 letter code of the village followed by serial numbers of each household) | \|  \|  \|  \|  \|  \|  \| \| --- \| --- \| --- \| --- \| --- \| --- \| | |
| --- | --- | --- | --- | --- | --- | --- | --- | --- |
| Date of survey  DD/MM/YY | \|  \|  \|  \|  \|  \|  \| \| --- \| --- \| --- \| --- \| --- \| --- \| | |
| Village Name: |  | |
| Interviewed by: |  | |
| **Section 1.1: Respondent’s Information**  *(Tick the answer and specify as required)* | |  |

|  | |  |
| --- | --- | --- |
| 1.1.1 | Name: |  |
| 1.1.2 | Age (Years): |  |
| 1.1.3 | Gender: | \| 1 \|  \| Male \| \| --- \| --- \| --- \| \| 2 \|  \| Female \| |
| 1.1.4 | Education level: | \| 1 \|  \| Not attended any school \| \| --- \| --- \| --- \| \| 2 \|  \| Attending/Attended Non-Formal Education \| \| 3 \|  \| Primary level \| \| 4 \|  \| High school \| \| 5 \|  \| Secondary level \| \| 6 \|  \| Graduate \| \| 7 \|  \| Buddhist studies \| \|  \|  \|  \| |

| **Section 1.2: Animal Population and Management**  *(Tick the answers and specify as required)*  1.2.1 **Number of cattle by breed** |
| --- |

*(Use breed specifications as per the livestock census guidelines)*

| **Animal species** | **Local** | **Crossbred** | **Purebred** |
| --- | --- | --- | --- |
| Cattle |  |  |  |

| 1.2.2 | Type of husbandry practice the farm follows: | \| 1 \|  \| Stall feeding \| \| --- \| --- \| --- \| \| 2 \|  \| Mix of stall feeding & free grazing \| \| 3 \|  \| Mix of stall feeding & tethered grazing \| \| 4 \|  \| All-time free grazing \| \| 5 \|  \| Mixture of the above practices \| |
| --- | --- | --- | --- | --- | --- | --- | --- | --- | --- | --- | --- | --- | --- | --- | --- | --- | --- |

| **Section 2.1: Knowledge and awareness of Ticks and Tick-Borne Diseases in cattle**  *(Tick the answers and specify as required)* |
| --- |

| 2.1.1 | Have you seen a tick? | \| 1 \|  \| Yes \| \| --- \| --- \| --- \| \| 2 \|  \| No \| |
| --- | --- | --- | --- | --- | --- | --- | --- | --- |
| 2.1.2 | If yes, where do you commonly find ticks?  *Respondents can have more than one answer.* | \| 1 \|  \| On the animals \| \| --- \| --- \| --- \| \| 2 \|  \| In the forests \| \| 3 \|  \| In the agriculture land \| \| 4 \|  \| In the pastureland \| \| 5 \|  \| All of the above \| |
| 2.1.3 | Which places do you think you find the ticks most commonly? | \| 1 \|  \| Warm places \| \| --- \| --- \| --- \| \| 2 \|  \| Cold places \| \| 3 \|  \| Both the places \| |
| 2.1.4 | Which season do you commonly see the ticks on the cattle? | \| 1 \|  \| Summer \| \| --- \| --- \| --- \| \| 2 \|  \| Winter \| \| 3 \|  \| Throughout the year \| |
| 2.1.5 | Where do you think the cattle get ticks from?  *Respondents can have more than one answer.* | \| 1 \|  \| From the forest \| \| --- \| --- \| --- \| \| 2 \|  \| From the grazing land \| \| 3 \|  \| From the bedding materials \| \| 4 \|  \| From fodder grasses \| \| 5 \|  \| Don’t know \| |
| 2.1.6 | Which type of cattle do you think gets mostly infested by the ticks? | \| 1 \|  \| Native breeds \| \| --- \| --- \| --- \| \| 2 \|  \| Exotic breeds \| \| 3 \|  \| Don’t know \| |
| 2.1.7 | Which of the cattle in the following do you think is most affected by tick infestation? | \| 1 \|  \| Adult \| \| --- \| --- \| --- \| \| 2 \|  \| Heifers \| \| 3 \|  \| Young calves \| \| 4 \|  \| Old cattle \| \| 5 \|  \| Don’t know \| |
| 2.1.8 | Which part of the body in animals do you commonly find ticks?  *Respondents can have more than one answer.* | \| 1 \|  \| Head region \| \| --- \| --- \| --- \| \| 2 \|  \| Neck region \| \| 3 \|  \| Groin and udder \| \| 4 \|  \| Chest and axillae \| \| 5 \|  \| Anus and perianal region \| \| 6 \|  \| Dewlap \| \| 7 \|  \| Feet (i.e., between or just above the hooves) \| \| 8 \|  \| Others (belly and limbs) \| |
| 2.1.9 | Do you think ticks always stay on the body of a cattle unless removed? | \| 1 \|  \| Yes \| \| --- \| --- \| --- \| \| 2 \|  \| No \| \| 3 \|  \| Don’t know \| |
| 2.1.10 | Have you also seen ticks on the body of other animals? | \| 1 \|  \| Yes \| \| --- \| --- \| --- \| \| 2 \|  \| No \|     *If no, go to the question 2.1.12* |
| 2.1.11 | If yes, in which species of animal did you see?  *Respondents can have more than one answer.* | \| 1 \|  \| Small ruminants (sheep and goats) \| \| --- \| --- \| --- \| \| 2 \|  \| Dogs \| \| 3 \|  \| Cats \| \| 4 \|  \| Equines \| \| 5 \|  \| Pigs \| \| 6 \|  \| Poultry \| \| 7 \|  \| Wild animals (including birds) \| |
| 2.1.12 | What do you think is the health and production impacts of tick infestation in cattle?  *Respondents can have more than one answer.* | \| 1 \|  \| Bloodsucking \| \| --- \| --- \| --- \| \| 2 \|  \| Bite wound \| \| 3 \|  \| Anorexia \| \| 4 \|  \| Loss of weight \| \| 5 \|  \| Fever \| \| 6 \|  \| Red or brown color urine \| \| 7 \|  \| Hide damage \| \| 8 \|  \| Loss of production \| \| 9 \|  \| Don’t know \| |
| 2.1.13 | Do you think cattle can get diseases from the ticks? | \| 1 \|  \| Yes \| \| --- \| --- \| --- \| \| 2 \|  \| No \| \| 3 \|  \| Don’t know \| |
| 2.1.14 | Have you heard of any tick-borne diseases in cattle? | \| 1 \|  \| Yes \| \| --- \| --- \| --- \| \| 2 \|  \| No \|   *If yes, move to the following question.* |
| 2.1.15 | If yes, from where did you hear about the tick-borne diseases in cattle?  *Respondents can have more than one answer.* | \| 1 \|  \| Farmers’ training program \| \| --- \| --- \| --- \| \| 2 \|  \| Livestock officials visiting farms \| \| 3 \|  \| Neighbors \| \| 4 \|  \| Family members \| \| 5 \|  \| Friends \| \| 6 \|  \| Media (including social media) \|   Any other, please specify? |

| \| **Section 2.2: Ticks and humans**  *(Tick the answers as required)* \| \| --- \| |
| --- | --- |

| 2.2.1 | Have you been bitten by ticks? | \| 1 \|  \| Yes \| \| --- \| --- \| --- \| \| 2 \|  \| No \|   *If No, move to question 2.2.3* |
| --- | --- | --- | --- | --- | --- | --- | --- | --- |
| 2.2.2 | What were the clinical signs of the tick bite?  *Respondents can have more than one answer.* | \| 1 \|  \| Pain and irritation \| \| --- \| --- \| --- \| \| 2 \|  \| Rash and swelling around the bite \| \| 3 \|  \| Fever and headache \| \| 4 \|  \| No symptoms \| |
| 2.2.3 | Do you think humans can get diseases from the tick bites? | \| 1 \|  \| Yes \| \| --- \| --- \| --- \| \| 2 \|  \| No \| \| 3 \|  \| Don’t know \| |

| **Section 3: Attitudes of the farmers**  *(Tick an appropriate column)* |
| --- |
| ***Start asking each question by “Do you agree?*** |

| **Attitude Questions on Likert Scale** | | **1** | | **2** | **3** | **4** | **5** | |
| --- | --- | --- | --- | --- | --- | --- | --- | --- |
| *3.1 Proper use of synthetic acaricides can reduce the incidences of tick infestation in cattle.* | | Strongly agree | | Disagree | No opinion | Agree | Strongly agree | |
| *3.2* *Risk of the tick infestation can be reduced by keeping the cattle always in the shed.* | | Strongly Disagree | | Disagree | No opinion | Agree | Strongly Agree | |
| *3.3* *Adopting good farm practices can reduce the risk of tick infestation (e.g., regular washing of floor, regular checking of animals, avoiding the use of bedding materials, etc.)* | | Strongly Disagree | | Disagree | No opinion | Agree | Strongly Agree | |
| **Section 4: Farmers’ Practices**  *(Tick the answers and specify as required)* | | | | | | | |  |
| 4.1 | What is the main purpose of rearing cattle in your household? | | \| 1 \|  \| Family consumption of products \| \| --- \| --- \| --- \| \| 2 \|  \| Income through the sale of products \| \| 3 \|  \| Income through the sale of animals \| \| 4 \|  \| As a source of manure \| \| 5 \|  \| Draft purpose \| \| 6 \|  \| Breeding purpose \|   Any other, please specify: | | | | | |
| 4.2 | What type of cattle shed do you have? | | \| 1 \|  \| Improved shed with CGI Sheet and concrete flooring \| \| --- \| --- \| --- \| \| 2 \|  \| Improved shed with CGI Sheet and wooden flooring \| \| 3 \|  \| Conventional Shed (built with local materials) \| \| 4 \|  \| Open-air tethering \| | | | | | |
| 4.3 | If your cattle shed’s floor is concrete, how often do you wash the flooring of your cattle shed? | | \| 1 \|  \| Daily \| \| --- \| --- \| --- \| \| 2 \|  \| Weekly \| \| 3 \|  \| Fortnightly \| \| 4 \|  \| Monthly \| \| 5 \|  \| Never \| | | | | | |
| 4.4 | Do you use bedding materials in your cattle shed? | | \| 1 \|  \| Yes \| \| --- \| --- \| --- \| \| 2 \|  \| No \| | | | | | |
| 4.5 | If yes, what type of bedding materials do you use in your cattle shed? | | \| 1 \|  \| Litter leaves \| \| --- \| --- \| --- \| \| 2 \|  \| Bracken fern \| \| 3 \|  \| Paddy straw \| \| 4 \|  \| Maize stover \| | | | | | |
| 4.6 | When do you mostly use bedding materials? | | \| 1 \|  \| Summer \| \| --- \| --- \| --- \| \| 2 \|  \| Winter \| \| 3 \|  \| Throughout the year \| | | | | | |
| 4.7 | Which animal health problems are most important to you?  *Select 3 of the problems* | | \| 1 \|  \| Mastitis \| \| --- \| --- \| --- \| \| 2 \|  \| Endoparasitism \| \| 3 \|  \| Milk fever \| \| 4 \|  \| Bacterial diseases (e.g., HS, BQ) \| \| 5 \|  \| Tick infestation \| \| 6 \|  \| Foot and mouth disease \| \| 7 \|  \| Plant poisoning \| | | | | | |
| 4.8 | What are the main purposes that make you visit the veterinary centers?  *Select 3 of the purposes* | | \| 1 \|  \| To receive livestock production inputs \| \| --- \| --- \| --- \| \| 2 \|  \| To receive medicines for sick animals \| \| 3 \|  \| To receive deworming drugs \| \| 4 \|  \| To receive acaricides \| \| 5 \|  \| To seek advice on farming practices. \| | | | | | |
| 4.9 | What control measures do you follow in your farm to address the problem of tick infestation in cattle?  *Respondents can have more than one answer.* | | \| 1 \|  \| Use acaricides \| \| --- \| --- \| --- \| \| 2 \|  \| Don’t let the animals out for grazing \| \| 3 \|  \| Follow rotational grazing \| \| 4 \|  \| Use homemade remedies \| \| 5 \|  \| Manually remove the ticks.  Adopt good farm practices \| | | | | | |
| 4.10 | What is the frequency of applying acaricides on your farm? | | \| 1 \|  \| Weekly \| \| --- \| --- \| --- \| \| 2 \|  \| Fortnightly \| \| 3 \|  \| Monthly \| \| 4 \|  \| Occasionally \| | | | | | |
| 4.11 | When you use acaricides, which method of application do you follow? | | \| 1 \|  \| Hand spraying \| \| --- \| --- \| --- \| \| 2 \|  \| Hand dressing \| \| 3 \|  \| Pour on \| \| 4 \|  \| Mixed of above practices \| | | | | | |
| 4.12 | How long does it take for ticks to fall off from the body of cattle after acaricide treatment? | | \| 1 \|  \| Ticks fall off within a few hours \| \| --- \| --- \| --- \| \| 2 \|  \| Ticks fall off within a day \| \| 3 \|  \| Ticks fall off within a few days \| \| 4 \|  \| Ticks fall off within a week. \| | | | | | |
| 4.13 | After applying acaricides or manual removal or brushing the infested animals, ticks fall off from the body of the cattle. What do you do with those ticks? | | \| 1 \|  \| Collect and burn \| \| --- \| --- \| --- \| \| 2 \|  \| Collect and throw it in the field \| \| 3 \|  \| Let it stay on the ground \| \| 4 \|  \| Flush it with running water \| | | | | | |
| 4.14 | Do you check your body for ticks after handling the tick-infested cattle? | | \| 1 \|  \| Always \| \| --- \| --- \| --- \| \| 2 \|  \| Sometimes \| \| 3 \|  \| Never \| | | | | | |
| 4.15 | Do you check your body for ticks after visiting the forests? | | \| 1 \|  \| Always \| \| --- \| --- \| --- \| \| 2 \|  \| Sometimes \| \| 3 \|  \| Never \| | | | | | |
| 4.16 | What do you do if the veterinary center has no acaricides? | | \| 1 \|  \| Manually remove \| \| --- \| --- \| --- \| \| 2 \|  \| Brush the animal \| \| 3 \|  \| Apply petroleum and kerosene products \| \| 4 \|  \| Apply *Zanthoxylum* mixture solution \| \| 5 \|  \| Apply salt solution \| \| 6 \|  \| Do nothing \| | | | | | |
| 4.17 | Other than treating tick infestation, where else do you think the acaricides can be used? | | \| 1 \|  \| Can be used as pesticides in the vegetable fields \| \| --- \| --- \| --- \| \| 2 \|  \| Can be used to get rid of insects, lice, mites, and bugs in homes \| \| 3 \|  \| Don’t know \| | | | | | |
